# Protective Effect of Gemfibrozil on Hepatotoxicity Induced by Acetaminophen in Mice: the Importance of Oxidative Stress Suppression

**DOI:** 10.15171/apb.2018.038

**Published:** 2018-06-19

**Authors:** Hojatolla Nikravesh, Mohammad Javad Khodayar, Masoud Mahdavinia, Esrafil Mansouri, Leila Zeidooni, Fereshteh Dehbashi

**Affiliations:** ^1^Toxicology Research Center, Ahvaz Jundishapur University of Medical Sciences, Ahvaz, Iran.; ^2^Department of Toxicology, School of Pharmacy, Ahvaz Jundishapur University of Medical Sciences, Ahvaz, Iran.; ^3^Cellular and Molecular Research Center, Department of Anatomical Sciences, School of Medicine, Ahvaz Jundishapur University of Medical Sciences, Ahvaz, Iran.

**Keywords:** Acetaminophen, Oxidative Stress, Gemfibrozil, Hepatoprotective, Mice

## Abstract

***Purpose:*** Gemfibrozil (GEM) apart from agonist activity at peroxisome proliferator-activated receptor-alpha (PPAR-α) has antioxidant and anti-inflammatory properties. Accordingly, the present study was designed to investigate the protective effect of GEM on acute liver toxicity induced by acetaminophen (APAP) in mice.

***Methods:*** In this study, mice divided in seven groups include, control group, APAP group, GEM group, three APAP groups pretreated with GEM at the doses of 25, 50 and 100 mg/kg respectively and APAP group pretreated with N-Acetyl cysteine. GEM, NAC or vehicle were administered for 10 days. In last day, GEM and NAC were gavaged 1 h before and 1 h after APAP injection. Twenty four hours after APAP, mice were sacrificed. Serum parameters include alanine aminotransferase (ALT), aspartate aminotransferase (AST) and liver tissue markers including catalase enzyme activity, reactive oxygen species (ROS), malondialdehyde and reduced glutathione (GSH) levels determined and histopathological parameters measured.

***Results:*** GEM led to significant decrease in serum ALT and AST activities and increase in catalase activity and hepatic GSH level and reduces malondialdehyde and ROS levels in the liver tissue. In confirmation, histopathological findings revealed that GEM decrease degeneration, vacuolation and necrosis of hepatocytes and infiltration of inflammatory cells.

***Conclusion:*** Present data demonstrated that GEM has antioxidant properties and can protect the liver from APAP toxicity, just in the same pathway that toxicity occurs by toxic ROS and that GEM may be an alternative therapeutic agent to NAC in APAP toxicity.

## Introduction

Liver diseases have become one of the main causes of morbidity and mortality in around the world and hepatotoxicity because of drugs appears to be the most common causal issue.^1-3^ APAP is one of the drugs that is commonly used to reduce fever and pain. This drug has few side effects at therapeutic doses^4,5^ but, overdose of APAP can cause liver toxicity.^6,7^ The mechanism of toxicity of this drug is because of the production of toxic metabolites, mitochondrial dysfunction and change the innate immune system.^8-11^ Liver is a main critical organ that metabolizes APAP in therapeutic doses in the form of glucuronidated and sulfated metabolites and the following metabolites excreted by urine.^5^ Approximately 2% excreted unchanged in the urine and 5% -10% through cytochrome P450 converted to the N-acetyl-p-benzoquinoneimine (NAPQI) which quickly reduced by GSH and excreted in the urine.^12^ NAPQI is a strong electrophile oxidizing agent that normally detoxified by GSH in the liver.^13-15^ But, in APAP overdose glucuronidation and sulfation pathways become to saturate and APAP metabolism by the cytochrome P450 produces a large amount of NAPQI that leads to rapid depletion of hepatic GSH levels.^16,17^ NAPQI causes impairs intracellular calcium homeostasis, increased cell permeability, reduced the integrity of cells membrane.^8,15^ It has been reported that released ATP, NAD and damage-associated molecular pattern (DAMP) from damaged hepatocytes can induced liver injury through P2X7 and Toll-like receptors in DAMP sensing cells.^18^ According the destructive effects of APAP in overdose and poisoning, new potential therapeutics for APAP overdose are routinely investigated in preclinical studies. Many of these studies have shown that pretreatment or simultaneous treatment with diverse agents can provide protection against APAP hepatotoxicity.^19,20^ Antioxidant agents have therapeutic potential in drug-induced toxicity.^21^ NAC is used as present treatment for APAP toxicity, by replacing cellular GSH to prevent of cell damage by NAPQI. Administration of NAC is critical to improving the clinical effect of APAP hepatotoxicity.^22^ Therefore, investigations on agents that have been therapeutic effect on APAP toxicity are attractive.

GEM is a fibrate drug that has approved for hyperlipidemia and commonly prescribed for lipid-lowering.^23,24^ The most important mechanism of fibrates is considered as agonists of PPAR-α.^25^ But, has recently reported that GEM has antioxidant and anti- inflammatory properties in diverse situations.^26-28^ In therapy for diabetes has shown that GEM increase the level of paraoxonase activity as an antioxidant enzyme which clean up free radicals and chelate metal ions;^27,29^ as well as can inhibit the pathways leading to inflammatory cytokine release,^30^ and also has shown that decreased atherosclerosis in diabetes via reduction in oxidative stress and inflammation.^31^ Others fibrates, have shown protective effects against inflammatory colitis,^32^ inflammatory heart in autoimmune myocarditis^33^ and neuroprotective effects against stroke.^34^ In contrast with these studies, there is other reports that GEM weather alone or in combination with statins is associated with the highest risk of myotoxicity as well as GEM causes to increase risk of cholelithiasis and cholestatic jaundice.^35,36^ Therefore, considering the importance of APAP toxicity and finding new therapeutic agents against its toxicity, GEM was selected according to its demonstrated properties. With these themes, the present study was designed to investigate the effects of GEM on acute hepatotoxicity induced by APAP in mice, mainly focusing on GEM antioxidant properties.

## Materials and Methods

### Animals

Experiments were performed on adult male NMRI mice aged 6-8 weeks old, weighing 23 to 27g. The animals were purchased from the Research Center and Experimental Animal House of Ahvaz Jundishapur University of Medical Sciences (AJUMS). They were retained in standard Plexiglas cages with room temperature (22-25 °C) with a 12 h light/dark cycle. During the study all animal have free access to food and water. The animals were acclimatized to the environment for three days before the initiation of research.

### Drugs and chemicals

APAP, thiobarbituric acid (TBA), 5,5'-dithiobis-2-nitrobenzoic acid (DTNB) and 2’,7’ –dichlorofluorescin diacetate (*DCFDA*) were purchased from Sigma-Aldrich (St Louis, Missouri, USA). GEM was donated by Dr. Abidi pharmaceutical company (Tehran, Iran), ALT and AST activity assay kits were obtained from (Pars Azmoon, Iran). Other chemicals used were of the highest grade available.

### Preparation of GEM and APAP solutions

GEM solution was prepared by wetting of GEM powder in glycerin and subsequently levigation in mortar by pestle with the addition of drinking water. APAP was dissolved in warm saline and administered (i.p.) at a dose of 400 mg/kg/10ml after 16 h overnight fasting to deplete hepatic GSH levels and thereby induce hepatotoxicity better.

### Experimental protocol

The mice were divided into seven groups (seven mice in each group).

- Received vehicle (10 ml/kg, p.o.) + a single dose of saline (10 ml/kg, i.p.)
- Received vehicle (10 ml/kg, p.o.) + a single dose of APAP (400 mg/kg/10ml, i.p.)
- Treated with GEM (25 mg/kg/10 ml, p.o.) + a single dose of APAP (400 mg/kg/10ml, i.p.)
- Treated with GEM (50 mg/kg/10 ml, p.o.) + a single dose of APAP (400 mg/kg/10ml, i.p.)
- Treated with GEM (100 mg/kg/10 ml, p.o.) + a single dose of APAP (400 mg/kg/10ml, i.p.)
- Treated with NAC (100 mg/kg/10 ml, p.o.) + a single dose of APAP (400 mg/kg/10ml, i.p.)
- Administered GEM (100 mg/kg/10 ml, p.o.) + a single dose of saline (10 ml/kg, i.p.)

The animals treated with GEM, NAC or vehicle orally once daily for 10 days and then received a single dose APAP. In tenth day, GEM and NAC were gavaged 1 h before and 1 h after APAP. At the end of the experimental period (24 hour after APAP administration), the animals were anaesthetized and blood samples were taken from heart. The serum was separated using centrifuge at 3500 rpm for 20 min for biochemical evaluation. Liver was removed and divided into two portions, one section homogenized for liver tissue biochemical tests and another part fixed in 10% formalin for histopathological assessments.

### ALT and AST activity of serum

Serum samples were used for measuring AST, ALT activity by using standard assay kits through auto analyzer (Biotechnical BT-3000 plus Chemistry Analyzer, Italy).

### Preparation of homogenized liver tissue

Liver tissue was homogenized 10% (w/v) with phosphate buffer (1 mM, pH 7.4), then centrifuged at 12,000×*g* for 30 min at 4°C. The supernatant was used for assessment.

### Determination of catalase activity in liver tissue

Catalase activity was determined by a slightly modified version. Accordingly, 500 µl of tris-HCl 0.05 mmol was added per one ml H_2_O_2_ and 50 µl of tissue extract (dissolved in 0.1 M, pH 7.2, phosphate buffer) were mixed and incubated for 10 min, then 500 µl Ammonium molybdate 4% was added and absorbance was measured at 410 nm. The result was expressed as U/g tissue.^37^

### Lipid peroxidation measurement in liver

Lipid peroxidation in the liver was measured based on the MDA level. The reaction of MDA with TBA produces a purple color with maximum absorbance at 532 nm.^38^ For this assessment, 1 ml supernatant was added to 2 ml TBA and placed in 100 °C for 15 min. After cooling, it was centrifuged (3000 RMP, 10 min) and the supernatant separated. Ultimately, the MDA level was reported as nmol/g tissue.^39^

### Evaluation of GSH level in liver tissue

The GSH level was measured using the Ellman’s reagent. Briefly, trichloroacetic acid 20% along with EDTA 1 mM was added to homogenate tissue. Then, it was centrifuged (10 min, 2000 rpm) and the isolated supernatant (200 μl) was added to 1.8 ml DTNB 0.1 mM. The absorbance was read at 412 nm by spectrophotometer and GSH level was reported as nmol/g tissue.^40^

### ROS level in liver tissue

DCFDA was used for ROS assay in the liver tissue. Cellular peroxides convert DCFDA into highly fluorescent DCF. Briefly, 10% liver homogenate was prepared in ice-cold Tris–HCl buffer 40 mM (pH 7.4). Then homogenate tissue was mixed with 1.25 mM DCFDA in methanol for ROS evaluation. All samples were incubated for 15 min in a water bath at 37 °C. Measurement was determined based on the intensity of fluorescence at 488 nm excitation and 525 nm emission wavelength using a fluorometer (Perkin-Elmer, LS-50 B, united Kingdom) and reported as fluorescence intensity unit (FIU).^41^

### Histopathological Assessments

For evaluation of microscopic changes, the liver was fixed in 10% formalin. Then, it was dehydrated through soaking in alcohol and xylol, respectively. Finally, after preparation of 5μ- tissue sections using rotary microtome, the hematoxylin and eosin (H&E) staining technique was performed. The histopathological changes were examined using light microscope.

### Statistical Analysis

Statistical analyses were performed with SPSS software, version 16.0 (SPSS, Inc., Chicago, IL, US). Continuous variables are expressed as mean ‏± SEM. Comparison of mean value was performed by one-way analysis of variance followed by with the Tukey’s post hoc test. Graphs were plotted using GraphPad Prism software. Statistical significance was set at p <0.05.

## Results

### Effect of GEM on serum activity levels of liver enzymes

**Figure 1:**
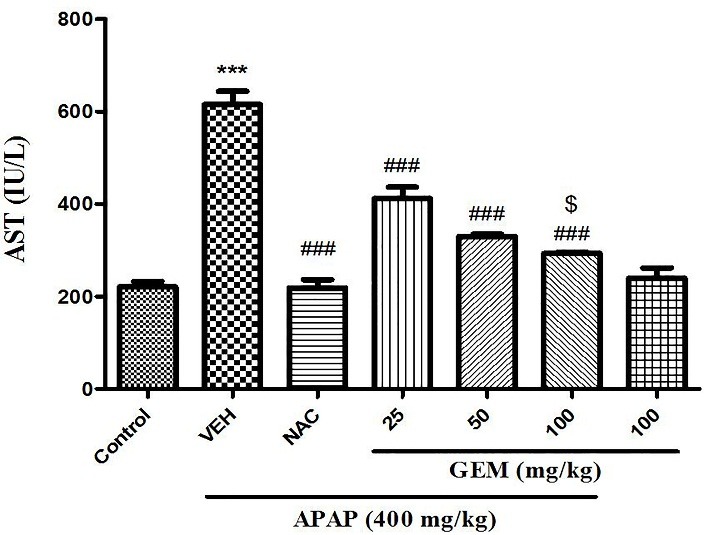
Effect of GEM on serum activity levels of AST in hepatotoxicity induced by APAP in mice. Data presented as Mean ± SEM (n=7). ^***^P<0.001 shows significant difference from control group, ^###^P<0.001 designates significant difference from APAP untreated group (VEH) and ^$^P< 0.05 indicates significant difference from GEM 25 and 50 mg/kg.

**Figure 2:**
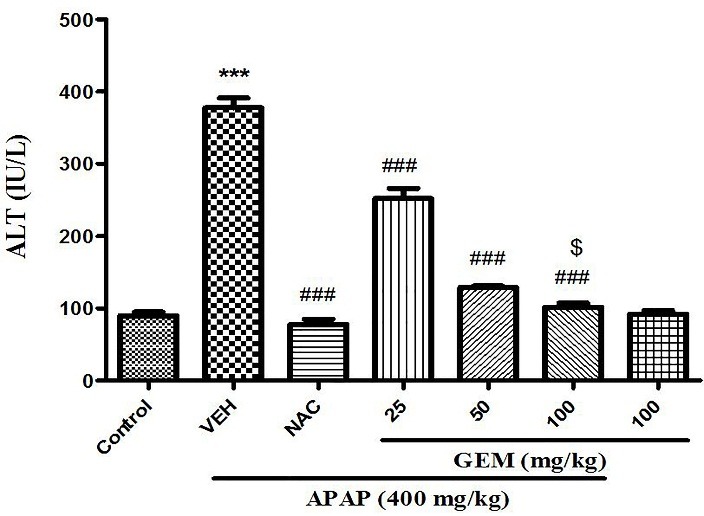
Effect of GEM on serum activity levels of ALT in hepatotoxicity induced by APAP in mice. Data presented as Mean ± SEM (n=7). ^***^P<0.001 designates significant difference from control group, ^###^P<0.001 defines significant difference from APAP untreated group (VEH) and ^$^P< 0.05 designates significant difference from GEM 25 and 50 mg/kg.

The results shown in Figure1 and Figure 2. The overdose of APAP can alter the serum liver enzymes activities‏. Serum ALT and AST levels indicate a measure of hepatic function. When mice were exposed to APAP, ALT and AST serum levels significantly increased compared with control group (p<0.001). GEM in doses of 25, 50 and 100 mg/kg could be significantly decrease ALT and AST serum activity levels compared with APAP untreated group (p<0.001). NAC also significantly decreased ALT and AST serum levels when compared with APAP group (p<0.001).

### Effect of GEM on liver tissue GSH level

**Figure 3:**
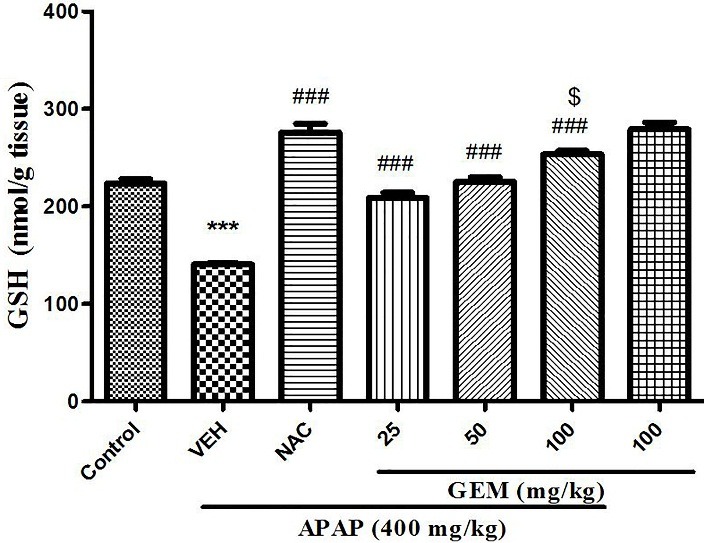
Effect of GEM on liver tissue levels of GSH in hepatotoxicity induced by APAP. Data presented as Mean ± SEM (n=7). ^***^P<0.001 designates significant difference from control group, ^###^P<0.001 shows significant difference from APAP untreated group (VEH) and ^$^P< 0.05 indicates significant difference from GEM 25 and 50 mg/kg.

As shown in (Figure 3) significant decrease in GSH level in liver was evident in APAP treated group when compared to control group (p<0.001). However, administration of GEM increased level of GSH when compared with APAP untreated group (p<0.001). And also NAC showed significant increase in GSH level when compared with APAP untreated group (VEH) (p<0.001).

### Effect of GEM on liver tissue thiobarbituric acid reactive substances (TBARS)

**Figure 4:**
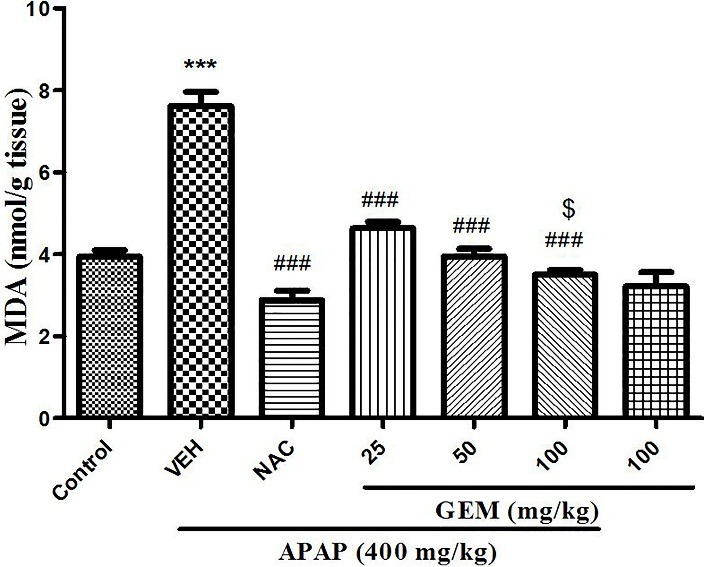
Effect of GEM on liver levels of MDA in hepatotoxicity induced by APAP. Data presented as Mean ± SEM (n=7). ^***^P<0.001 designates significant difference from control group, ^###^P<0.001 indicates significant difference from APAP group and ^$^P< 0.05 designates significant difference from GEM 25 and 50 mg/kg.

The results of lipid peroxidation in (Figure 4) showed that liver tissue MDA level in APAP treated group significantly increased when compared to control group (p<0.001). However, administration of GEM in used doses in treated animals suppressed MDA level in the liver tissue when compared with APAP group (p<0.001). And also NAC showed significant decrease in liver tissue MDA level when compared with APAP group (p<0.001).

### Effect of GEM on liver tissue catalase activity

**Figure 5:**
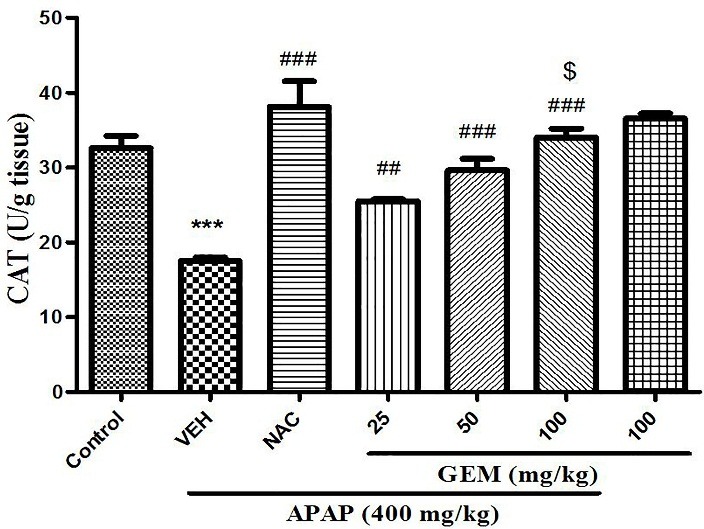
Effect of GEM on liver activity of catalase in hepatotoxicity induced by APAP. Data presented as Mean ± SEM (n=7). ^***^P<0.001 designates significant difference from control group, ^##^P<0.01 and ^###^P<0.001 show significant difference from APAP untreated group and ^$^P< 0.05 indicates significant difference from GEM 25 and 50 mg/kg.

As shown in (Figure 5) catalase activity in APAP treated group significantly decreased when compared to control group (p<0.001). However, administration of GEM in doses of 50 and 100 mg/kg (p<0.001) and in dose 25 mg/kg (p<0.01 elevated catalase activity of liver tissue when compared with APAP untreated group. NAC showed significant increase in liver tissue catalase activity when compared with APAP group (p<0.001).

### Effect of GEM on liver tissue ROS level

**Figure 6:**
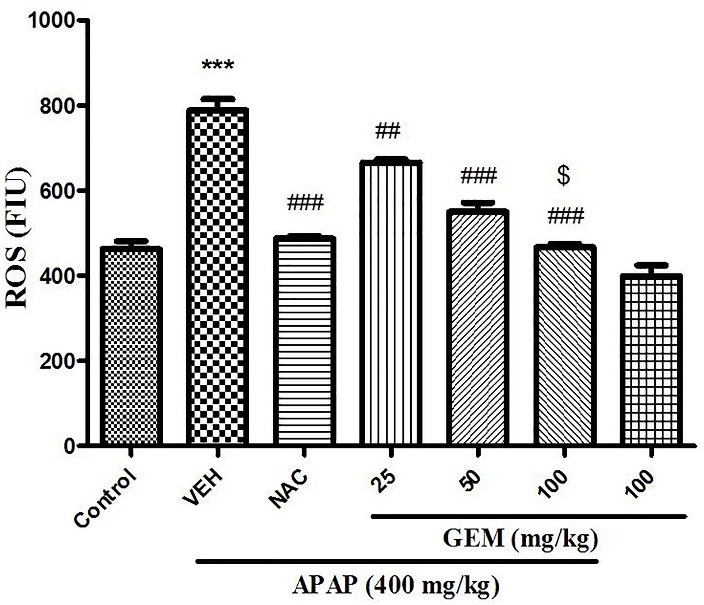
Effect of GEM on liver tissue levels of ROS in hepatotoxicity induced by APAP. Data presented as Mean ± SEM (n=7). ^***^P<0.001 designates significant difference from control group, ^##^P<0.01 and ^###^P<0.001 indicate significant difference from APAP group and ^$^P< 0.05 indicates significant difference from GEM 25 and 50 mg/kg.

The results in (Figure 6) indicated that ROS intensity in APAP treated group significantly enhanced when compared to control group (p<0.001). However, administration of GEM in doses 50 and 100 mg/kg (p<0.001) and in dose 25 mg/kg (p<0.01) decreased intensity of ROS in the liver tissue when compared with APAP group. Furthermore, NAC showed significant increase in liver tissue ROS intensity when compared with APAP group (p<0.001).

### Histopathological analysis

**Figure 7:**
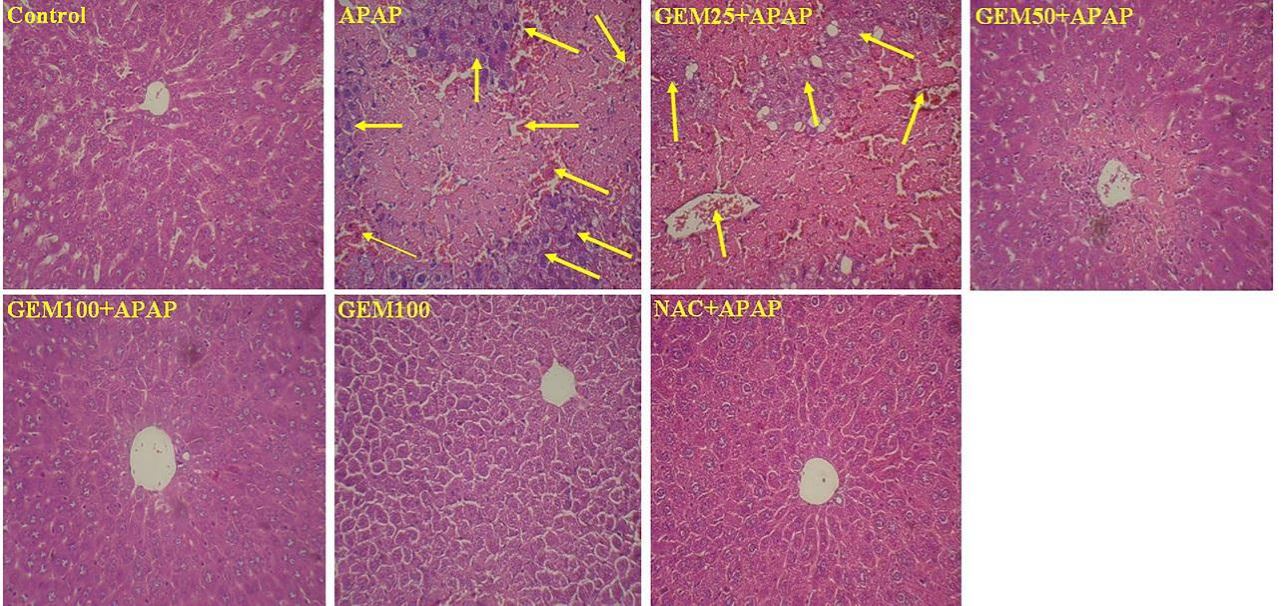
Photomicrograph sections of liver tissues in hepatotoxicity induced by APAP and protective effect of GEM (original magnification: x40). The mice livers: Control; Acetaminophen (APAP); APAP pretreated with GEM 25 mg/kg (GEM25 +APAP); APAP pretreated with GEM 50 mg/kg (GEM50 +APAP); APAP pretreated with GEM 100 mg/kg (GEM100 +APAP); GEM 100 mg/kg (GEM100); APAP pretreated with N-Acetyl cysteine 100 mg/kg (NAC +APAP).

Histopathological findings in control liver tissue indicated that hepatocyte cells, lobules and sinusoid status are normal and do not show any damage. But, in APAP group degeneration of hepatocytes, vacillation of vessels, the necrosis of hepatocytes, infiltration of inflammatory cells, and dilated sinusoids were observed (Figure 7). In mice that received APAP plus GEM 25 mg/kg liver damage can be observed but little improvement has been found. Furthermore, in mice that received APAP plus GEM 50 mg/kg, tissue damages and vacillation of vessels significantly improved, sinusoid has been almost normalized and only a small amount of damage in the form of infiltration of inflammatory cells can be seen around the central vein. Mice that received APAP plus GEM 100 mg/kg, liver tissues are normal and there was no serious damage, sinusoids and hepatic cells similar to the control group. In addition, in mice that received APAP plus NAC 100 mg/kg and mice that only received GEM 100 mg/kg, lobules of the liver tissues, cells, and sinusoids are normal and pathological changes were not observed (Figure 7). Semi quantitative scoring of hepatic damage and inflammation induced by APAP and improvement by GEM shown in Table 1.

**Table 1:** Semi quantitative scoring of histopathological examination of liver tissues. (-) indicates normal and (+) indicates mild, (++) indicates moderate, (+++) indicates severe, (++++) indicates extremely severe toxicity and damage.

**Groups** **Symptoms**	**Control**	**APAP**	**GEM25 + APAP**	**GEM50 + APAP**	**GEM100 + APAP**	**GEM100**	**NAC + APAP**
Hepatocyte degeneration	-	++++	++++	++	-	-	-
Hepatocyte vacuolation	-	+++	+++	+	-	-	-
Necrosis in hepatocyte	-	++++	++++	++	-	-	-
Infiltration of inflammatory cells	-	++	++	++	+	-	-
Dilated sinusoids	-	++++	+++	-	-	-	-

## Discussion

In the present study, the liver cell injury by a single dose of APAP was associated with significant increase in serum ALT and AST activities while GEM caused to significant decrease in serum ALT and AST activities. In addition, NAC at the dose 100 mg/kg significant attenuates increasing in activities of these two enzymes due to APAP intoxication. Increasing serum activity levels of transaminases (AST and ALT) have been indicated to liver tissue dysfunctions because these are normally located in the cytoplasm and leakage occurs into the circulation after cellular damage. Normalization serum activity levels of these two enzymes by GEM indicating protection of liver cells. APAP induced injury by its free radical metabolites^12,16^ and the protection of liver via antioxidant activity or inhibition of the generation of free radicals are important in the protection against APAP-induced liver injury.^42^ Furthermore, pretreatment of mice with GEM 25, 50 and 100 mg/kg against APAP–induced acute hepatotoxicity indicates that GEM ameliorates liver damage via augmentation of liver antioxidant function. Suppression of oxidative stress confirmed by catalase activity and ROS level as well as GSH and MDA levels in liver. It has been shown that different doses of GEM have protective effect in brain damage^43^ as well as indicated that GEM has protective effect against the acute restraint stress-induced disturbances in hippocampus in male rat^44^ and also demonstrated that GEM causes to decrease atherosclerosis in diabetes via reduction in oxidative stress and inflammation.^31^

GEM possess profound anti-inflammatory and antioxidant activity which, antioxidant properties dependent or independent to PPAR α/β receptors.^27^

In APAP toxicity, oxidative stress plays a key role in APAP-induced hepatotoxicity. Increased ROS levels leads to thiol oxidation that as a result decrease cellular GSH level and reduce activity of catalase enzyme function.^45^ GSH is the major intracellular defense molecule against ROS and prevents of oxidative stress in cells. In APAP toxicity GSH causes to detoxifying NAPQI and prevents of oxidative damage to hepatocytes.^45-47^ Therefore intracellular GSH levels are crucial in protecting from the toxic metabolite of APAP.

**Figure 8:**
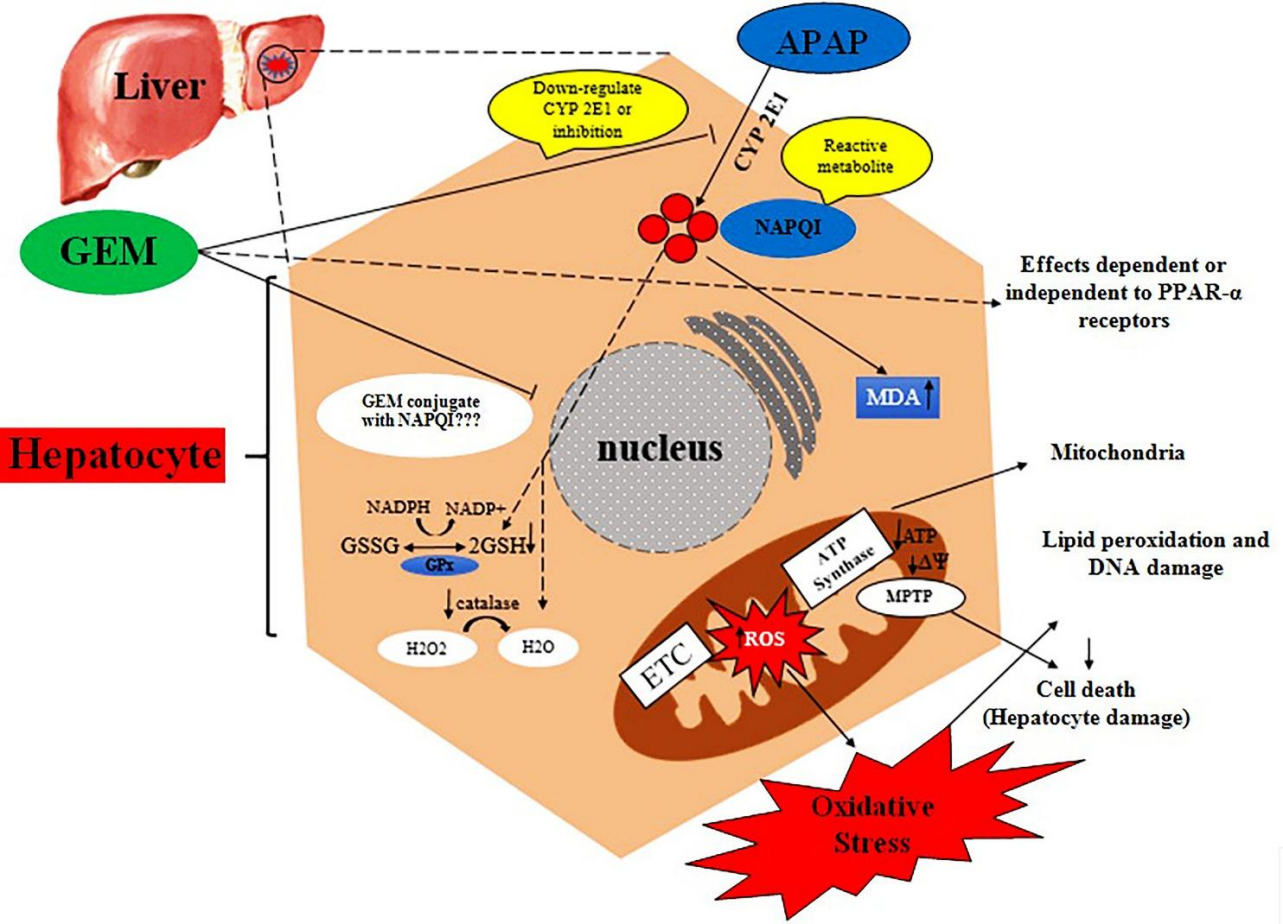
Graphical abstract represented regarding possible effects of GEM on liver damage induced by APAP. GEM can inhibit cytochrome P450 2E1 thus prevention the formation of NAPQI and/or that reacts with NAPQI and inhibits the action of NAPQI to liver macromolecules. GEM restore hepatocyte GSH content and catalase activity and inhibits the formation of ROS and MDA which this antioxidant properties dependent or independent to PPAR α receptors. (ETC: electron transport chain; MPTP: Mitochondrial permeability transition pore)

Present data indicated significant decrease in GSH levels 24 hours after APAP exposure. In confirming, others studies reported a significant decrease in GSH level following APAP administration.^48,49^ In our study GEM at used doses caused a significant enhance in GSH level compared with APAP group. However, pretreatment with GEM showed an increase in GSH levels that likely inhibits formation of NAPQI or directly reacts with NAPQI and/or prevents of its reaction with proteins (Figure 8).

ROS level increased 24 hours after APAP administration in liver. In APAP overdose, increase in NAPQI level is the main cause that leads to formation of superoxide anion (O2∙-) and hydrogen peroxide (H2O2). GEM in all used doses decreased ROS level and this implies antioxidant properties of GEM (Figure 8).

Antioxidant activity of catalase is important for the removal of ROS. It has been suggested that the tissue activity of catalase may reflect ROS levels.^47^ Catalase enzyme activity significantly decreased in liver 24 hours after APAP administration and GEM in all used doses increased catalase activity in APAP treated mice. This reveals increasing GSH level and alleviation ROS level by GEM (Figure 8).

ROS attacks lipid membranes and its result lipid peroxidation, impaired membrane function and generation of MDA that MDA is the end-product of lipid peroxidation and can be a indicator of oxidative stress.^50^ Consequently, ROS can be calculate indirectly with the amount of MDA and the levels of some antioxidant enzyme activities like catalase in tissue.^51^ In previous studies fibrates showed that decreased MDA production in diabetic rats,^52^ and also pretreatment with fibrates caused to reduce in MDA level and depletion of endogenous antioxidants.^53^

In our study, the MDA level significantly enhanced in liver tissue in mice exposed to APAP and pretreatment with GEM in all used doses significantly decrease MDA level which suggests GEM may be effective in the prevention of lipid peroxidation formation (Figure 8).

Our findings indicated that GEM significantly inhibited the acute hepatotoxicity induced by high doses of APAP (400 mg/kg) in mice, as shown by a decrease in serum liver enzyme activities AST, ALT levels and also MDA and ROS levels and also increased in GSH level and catalase activity. Moreover, the liver tissue morphology and histopathology findings confirmed the protective activity of this drug against the APAP-induced hepatotoxicity. It is evident by the reversal of degenerative of hepatocytes, vacillation of vessels, the necrosis of hepatocytes, infiltration of inflammatory cells, and dilated sinusoids in hepatic parenchyma by GEM administration. Although this protective effect was dose-dependent, there was no significant difference between doses of 25 and 50. But, according to the results of biochemical and pathological, our study indicates that the highest protective effect is related to GEM at the dose 100 mg/kg. Animal received dose 100 mg/kg GEM and also NAC did not have any differences with control group based on biochemical and histopathological findings and did not cause liver damage. It has been reported that induction of PPAR-α attenuates the extent of oxidative stress in steatohepatitic mice receiving APAP and has hepatoprotective effect due to high repair of liver tissue but not due to suppression of APAP bioactivation.^54^ However, application of therapeutic agents before and after APAP can associated with a chance of improvement.

## Conclusion

In conclusion, we have shown that GEM has therapeutic effect along with antioxidant activity in APAP-induced hepatotoxicity. Our results propose that GEM with antioxidant properties can protect the liver from the oxidative damage induced by toxic chemicals. GEM may be an alternative therapeutic agent to NAC in APAP toxicity. However, more investigations are needed to realize the mechanism of GEM effects especially modulation and activity of PPAR-α receptors.

## Acknowledgments

This paper is issued from a Master of Science thesis in Toxicology of Hojatolla Nikravesh and was financially supported by Toxicology Research Center of Ahvaz Jundishapur University of Medical Sciences, Ahvaz, Iran. (Grant No: TRC-9406).

## Ethical Issues

The study was performed according to the Animal Ethics Committee guidelines of AJUMS for the use of experimental animals (code of ethics: IR.AJUMS.REC.1394.602).
